# Modulation of endoplasmic reticulum stress–induced insulin resistance by the low-carbohydrate high-fat ketogenic diet

**DOI:** 10.3389/fnut.2025.1704597

**Published:** 2026-01-06

**Authors:** Basmah M. Eldakhakhny, Fatma M. Ghoneim, Yousef M. Almoghrabi, Ghada Ajabnoor, Faisal Alandejani, Salwa M. Abo El-Khair, Salma A. Elsamanoudy, Taghreed Shamrani, Moaaz A. Siddiqui, Ayman Z. Elsamanoudy

**Affiliations:** 1 Clinical Biochemistry Department, Faculty of Medicine, King Abdulaziz University, Jeddah, Saudi Arabia; 2 Food, Nutrition, and Lifestyle Research Unit, King Fahd for Medical Research Centre, King Abdulaziz University, Jeddah, Saudi Arabia; 3 Physiological Sciences Department, Fakeeh College for Medical Sciences, Jeddah, Saudi Arabia; 4 King Fahd Medical Research Center, Regenerative Medicine Unit, King Abdulaziz University, Jeddah, Saudi Arabia; 5 Medical Biochemistry and Molecular Biology Department, Faculty of Medicine, Mansoura University, Mansoura, Egypt; 6 Mansoura-Manchester Medical Program for Medical Education, Faculty of Medicine, Mansoura University, Mansoura, Egypt; 7 Faculty of Medicine, King Abdulaziz University, Jeddah, Saudi Arabia

**Keywords:** endoplasmic reticulum stress, endoplasmic reticulum stress response, inflammatory response, insulin resistance, low-carbohydrate high-fat diet, metabolic disorders

## Abstract

This review aimed to investigate the relationship between endoplasmic reticulum (ER) stress, insulin resistance, and the potential mitigating effects of a low-carbohydrate, high-fat diet, Ketogenic diet (LCHF-KD). A detailed literature search using databases to achieve a comprehensive overview. The keywords of the search were “endoplasmic reticulum stress,” “insulin resistance,” “metabolic syndrome,” and “low carbohydrate-high fat diet, molecular mechanism, Biochemical effects, Metabolic effects, Signaling pathways.” Insulin resistance is a metabolic disorder characterized by decreased cell sensitivity to insulin, resulting from the interplay between genetic and environmental factors. It can act as both a result and trigger of uncontrolled endoplasmic reticulum stress. This condition is associated with several disruptions, including impaired endoplasmic reticulum-mitochondrial transport, disordered signaling pathways, macrophage dysfunction, autophagy, immune function, inflammatory responses, dysregulation of antioxidant responses, and altered expression of genes involved in the endoplasmic reticulum stress response. LCHF-KD has been shown to alleviate insulin resistance associated with endoplasmic reticulum stress. Finally, it is concluded that ER stress plays a crucial role in the development of insulin resistance and metabolic diseases, including type 2 diabetes and obesity. Therapeutic strategies, including chemical chaperones and unfolding protein response (UPR) modulators, were used to alleviate ER stress. Dietary interventions, such as the low-carbohydrate, high-fat ketogenic diet (LCHF-KD), also reduce ER stress and improve metabolic health by modulating inflammation and oxidative stress. Combining these with conventional dietary therapies and personalized medicine approaches may enhance treatment outcomes and prevent the progression of metabolic disorders.

## Introduction

Insulin resistance (IR) is a pathological condition characterized by the diminished ability of cells to uptake glucose in response to insulin (1). IR is characterized by combined hyperinsulinemia and chronic hyperglycemia and is recognized as the pathogenic mechanism of type 2 diabetes mellitus (T2DM) (2). Furthermore, IR is linked to metabolic syndrome. The Adult Treatment Panel III (ATP III) of the National Cholesterol Education Program defines metabolic syndrome as the presence of three or more of the following five specific clinical criteria. These include abdominal obesity, hypertriglyceridemia, reduced HDL cholesterol, hypertension, and hyperglycemia. This definition underscores the role of insulin resistance and central obesity as central features of metabolic syndrome and highlights the associated risk for type 2 diabetes and cardiovascular disease (3). The high global incidence of IR and its pathogenesis has substantial risk factors for cardiovascular diseases, underscoring its impact on morbidity and mortality (4).

The pathophysiology of IR involves complex biochemical and molecular pathways, including the impairment of insulin receptor signaling (5), alterations in glucose transporter (GLUT) expression (6), endoplasmic reticulum (ER) stress, and chronic inflammation (7). Beyond impaired glucose homeostasis, IR also causes abnormalities in lipid metabolism. It exacerbates dyslipidemia by increasing plasma-free fatty acids, hypertriglyceridemia, and the formation of small dense low-density lipoprotein cholesterol (sdLDL-C), which contributes to the development of atherosclerosis (8).

The Endoplasmic Reticulum (ER) is the largest organelle of the eukaryotic cell and is central for protein folding, maturation, and trafficking (9). Under conditions of cellular stress, the amount of newly synthesized proteins decreased to allow time for protein folding in the ER. This folding delay can lead to the accumulation of misfolded and unfolded proteins within the ER lumen, a phenomenon known as endoplasmic reticulum (ER) stress (9). In response, several signaling pathways are activated collectively as the unfolded protein response (UPR), which aims to restore endoplasmic reticulum homeostasis by enhancing protein folding capacity, decreasing protein synthesis, and degrading misfolded proteins (10, 11).

Chronic ER stress is associated with the development of a variety of pathological conditions, including neurodegenerative diseases (12), cancer (13), and metabolic disorders (14). In particular, ER stress interferes with insulin signaling pathways, contributing to the development of metabolic diseases and insulin resistance (15, 16).

The low-carb, high-fat diet (LCHF), commonly known as the ketogenic diet (KD), is adopted for its distinctive approach to weight loss and health enhancement. The low-carb, high-fat ketogenic diet (LCHF-KD) is characterized by its low-carbohydrate, high-fat, and moderate-protein composition. Typically, carbohydrates provide less than 10% of total energy intake, fats contribute approximately 70–75%, and proteins account for about 15–20%. This macronutrient distribution promotes a metabolic shift from glucose to fat utilization, leading to increased ketone body production and improved metabolic flexibility (17). The underlying rationale behind the ketogenic diet is its ability to shift the body’s primary energy source from glucose, derived from carbohydrates, to ketones, which are produced through the oxidation of fat. This metabolic adaptation takes several days to weeks. During this period, the body undergoes considerable modifications in its energy metabolism (18).

The LCHF-KD offers several health benefits, including weight loss, improved glycemic control, and enhanced neurological function, particularly in conditions such as epilepsy, Alzheimer’s disease, and Parkinson’s disease. These health benefits are mainly attributed to the neuroprotective properties of ketone bodies (19). The LCHF-KD is a potential intervention to improve insulin resistance (20) and endoplasmic reticulum (ER) stress (21). The impact of the LCHF-KD on ER stress and insulin resistance is based on the alteration of substrate availability and utilization that, in turn, alleviates ER stress and enhances insulin signaling pathways (20, 21). Moreover, ketone bodies generated during the LCHF-KD exhibit anti-inflammatory and antioxidant effects, which improve mitochondrial function and thereby reduce ER stress while enhancing insulin sensitivity (22).

Despite significant progress in understanding the complex relationship between ER stress and insulin resistance (IR), many aspects remain unclear. Critical unanswered questions involve identifying the specific molecular triggers of ER stress in insulin-dependent tissues and understanding how these triggers differ among individuals with varying genetic backgrounds and lifestyles. Additionally, the effects of particular nutrients versus overall dietary patterns on ER stress and insulin resistance are not fully understood, indicating a need for more detailed research into how individual dietary components impact these pathways, particularly LCHF-KD. Furthermore, there is a significant gap in understanding the variability in susceptibility to insulin resistance and related metabolic disorders in response to ER stress, as well as the influence of genetic factors, epigenetic changes, and differences in microbiome composition. So, the primary research question for this review is: “How does ER stress contribute to insulin resistance in metabolic syndromes, and can LCFH-KD mitigate these effects?

This review article aims to provide a comprehensive examination of the role of ER stress in the development and progression of IR, with a particular focus on the underlying molecular mechanisms that link ER stress to impaired insulin signaling. It explores how ER stress contributes to metabolic dysfunctions commonly observed in insulin resistance, metabolic syndrome, and their related comorbidities. Moreover, the review critically evaluates the potential protective effects of a LCHF-KD in mitigating ER stress-induced insulin resistance. By synthesizing evidence from experimental and clinical studies, it investigates how alterations in dietary composition, specifically carbohydrate restriction and increased fat intake, can influence ER stress responses and thereby enhance insulin sensitivity. This integrated analysis underscores the importance of nutritional interventions in modulating cellular stress mechanisms and enhancing metabolic outcomes. Furthermore, this review contributes to the evolving fields of nutrition, metabolism, and personalized medicine by providing insights that support the development of personalized dietary strategies for preventing and managing insulin resistance and associated metabolic disorders.

## Methods

In this scoping review, we aimed to investigate the relationship between endoplasmic reticulum stress, insulin resistance, and the potential mitigating effects of a LCHF-KD. The authors conducted a comprehensive literature search using databases such as PubMed, Scopus, Web of Science, and Google Scholar to gain a detailed overview. The keywords of the search were “endoplasmic reticulum stress,” “insulin resistance,” “metabolic syndrome,” and “low carbohydrate-high fat diet, molecular mechanism, Biochemical effects, Metabolic effects, Signaling pathways.” The search included peer-reviewed research articles published within the last 20 years (2005–2025) to ensure the inclusion of the most current data.

The inclusion criteria encompass randomized controlled trials, cohort studies, case–control studies, experimental animal studies, and cross-sectional studies that investigate the interplay between ER stress and insulin resistance in IR- related conditions, such as metabolic syndrome, type 2 diabetes, and obesity. Research investigating the impact of dietary interventions, specifically LCHF-KD intake, was also included. Only peer-reviewed articles published in English are used in the current review to ensure scientific validity. In contrast, our exclusion criteria included reviews, opinion pieces, editorials, and non-empirical reports that do not contribute original data. Studies that did not focus on the impact of LCHF-KD or addressed other dietary interventions were also excluded, as were those carried out exclusively on animal models with no clinical implications.

The selection of articles involved a two-step process: an initial screening of titles and abstracts to discard studies that did not meet the criteria, followed by a thorough review of full texts to assess their relevance based on predefined inclusion criteria.

Data extraction and information collection, including publication year, methodology, and key findings related to our research question, were conducted and analyzed to assess the relationship between ER stress and metabolic health outcomes, particularly in the context of LCHF-KD interventions. Then, the results from the various studies were integrated to outline the connections between ER stress, insulin resistance, and the effects of LCHF-KD.

**Figure 1:**
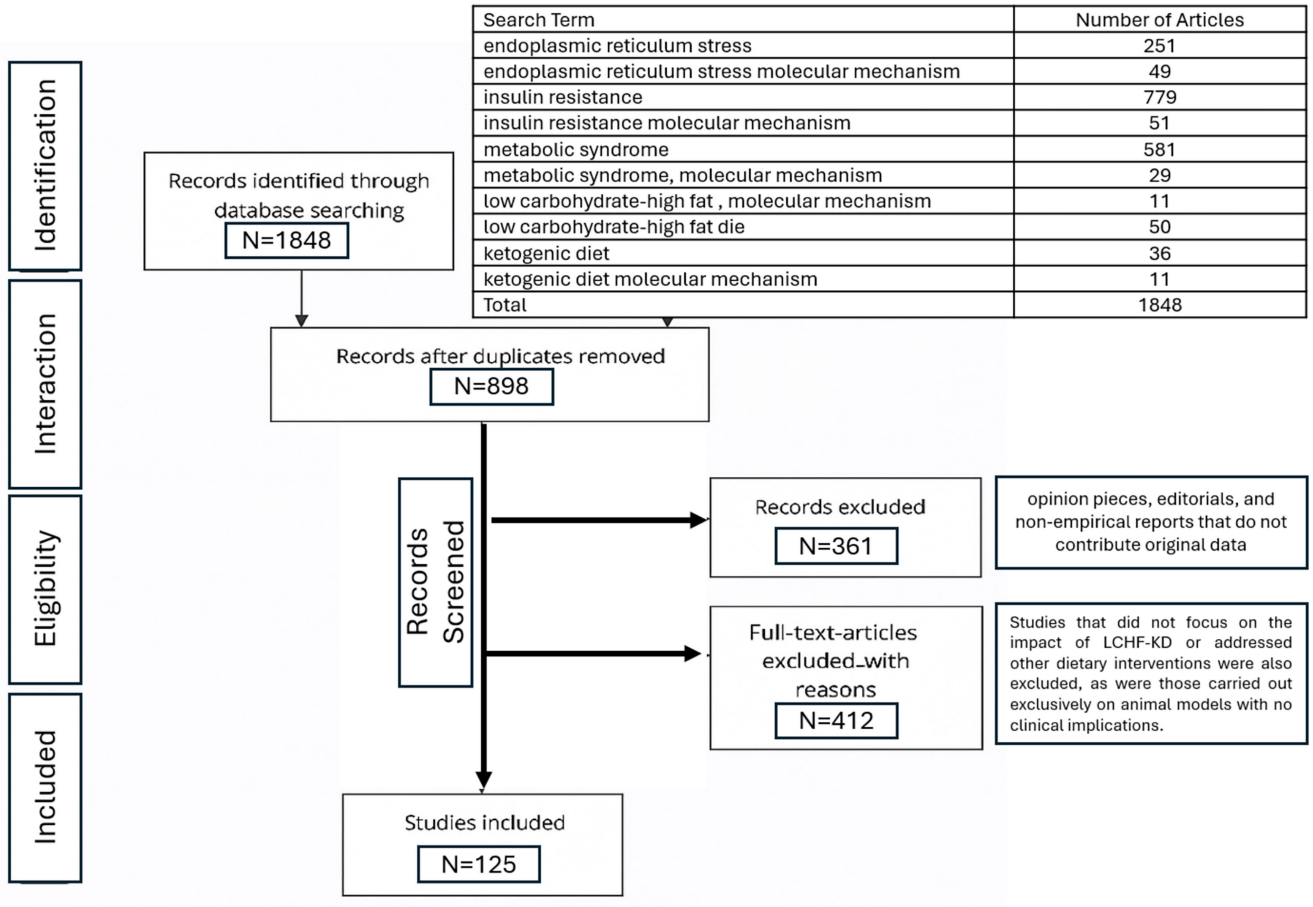
Study selection flow of the scoping review process: PRISMA-ScR flow chart.

The current scoping review was conducted in accordance with the PRISMA-ScR (Preferred Reporting Items for Systematic Reviews and Meta-Analyses Extension for Scoping Reviews) guidelines (23). A standardized data charting tool was employed to extract essential information from each study, including study design, participant characteristics, interventions or exposures, outcomes, and key results. Data extraction was independently conducted by two authors, with any disagreements resolved through discussion. The results were synthesized through narrative analysis to identify existing patterns and gaps in the literature. Since the review was based exclusively on previously published studies and did not involve the use of individual patient data, ethical approval was not required under institutional policies. The PRISMA-ScR flow chart is presented in Figure 1.

### Understanding insulin resistance

**Figure 2:**
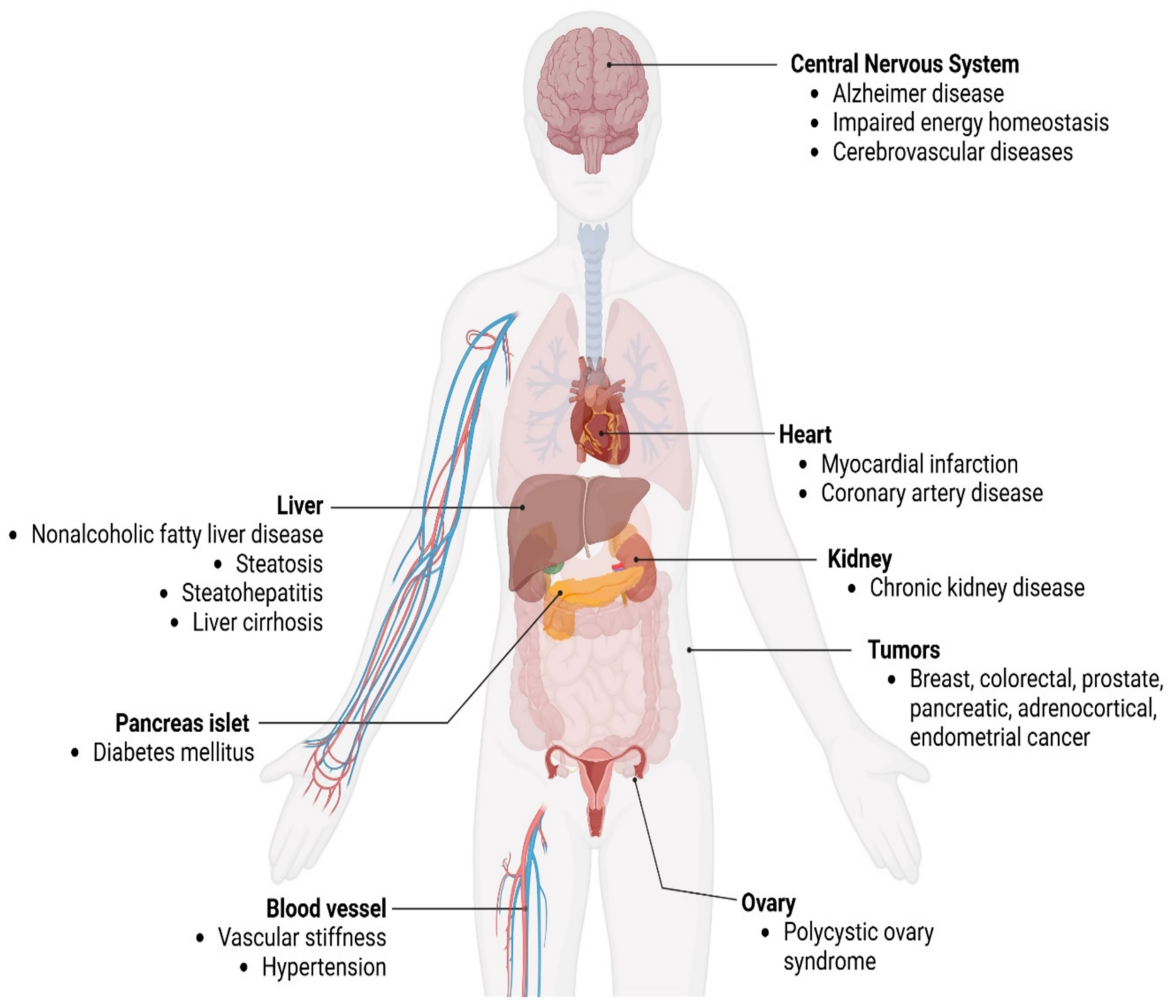
Chronic metabolic diseases may be induced by insulin resistance, created in BioRender. Eldakhakhny, B. (2025), https://BioRender.com/r39m848.

IR is a metabolic disorder characterized by reduced cellular sensitivity to insulin (6). As a result of this condition, there is an inadequate response to normal insulin levels, causing a higher secretion of insulin (hyperinsulinemia) and glucose (hyperglycemia) in the bloodstream (24). Hyperinsulinemia is thus considered a compensatory mechanism for suppressed insulin action (25). IR is also characterized by insufficient insulin-mediated blood glucose regulation, dyslipidemia, and increased adipocyte lipolysis. These associated metabolic conditions are collectively known as insulin resistance syndrome or metabolic syndrome (26). Moreover, IR plays a key pathological role in many leading mortality diseases, such as T2DM, cardiovascular and cerebrovascular diseases (27). Figure 2 demonstrates the most common chronic metabolic diseases that IR may induce.

### The pathogenesis of insulin resistance

The pathogenesis of IR mainly results from the interaction between genetic/hereditary and environmental factors. This interaction disrupts internal environment homeostasis, exhibiting as chronic subclinical inflammation, persistent hypoxia, lipotoxicity, immune dysregulation, and metabolic dysfunctions (26).

### Genetic/hereditary factors

The incidence of IR is frequently observed in families and certain ethnic groups, suggesting an important role for genetic and hereditary factors in the development of this condition. However, the complete genetic constitution of this disease has not yet been fully elucidated (28). Nevertheless, several genetic factors associated with IR have been identified, including mutations in insulin-related genes that produce mutant human insulins, genetic defects in the insulin signaling system, and genetic defects related to substance metabolism (29). The most common genetic mutations and their associations are listed in Table 1.

**Table 1:** The most common genetic mutations and their associations to insulin resistance.

Gene/protein/family	Mutation/association	SNP	Impact on insulin resistance (IR)	Reference
Type 4 glucose transporter (GLUT4)	Mutations	Not specifically identified	Impaired glucose uptake by skeletal muscle and adipose tissue IR	(106)
Glucokinase	Mutations	rs1799884	Affect glucose metabolism IR	(107)
Peroxisome proliferator-activated receptor (PPAR) nuclear receptor family	Mutations	rs1801282 (PPARG Pro12Ala)	Impaired glucose and lipid metabolism IR	(108)
Lipid metabolic pathways: leptin, adiponectin, resistin (and their receptors)	Mutations	rs1137101 (LEPR), rs2241766 (ADIPOQ), (rs1862513) RETN -420C > G	It affects glycolipid metabolism, development, and IR	(109, 110)
Peroxisome proliferator-activated receptors [(PPAR)α, γ, δ]	Mutations	rs1800206 (PPAR α), rs1801282 (PPAR γ), rs2016520& rs1053049 (PPAR) δ	Impaired glucose and lipid metabolism IR	(111)
AKT2/PKBβ (in cultured cells)	Mutation	rs3730051	Disrupts insulin signaling and inhibits AKT/PKB co-expression	(112)
Protein tyrosine phosphatase N1 (PTPN1)	Relationship mediated by regulatory region variants	rs3787348, rs6067484	Revealed through high-throughput genetics	(113)
TCF7L2 rs7903146 (T allele)	Healthy carriers	rs7903146	Increases insulin secretion, leads to impaired β-cell function, and increases the risk of T2DM	(114)

### Environmental, health, and lifestyle factors

Besides genetic factors, several interconnected environmental and lifestyle-related factors contribute to the development of insulin resistance (IR). Obesity is considered the most prominent. Central or visceral obesity impairs insulin function by disrupting glucose uptake and increasing hepatic glucose output. The accumulation of adipose tissue induces systemic insulin resistance (IR) through endocrine dysregulation and chronic inflammation. Elevated levels of free fatty acids (FFAs), tumor necrosis factor-alpha (TNF-α), and interleukin-6 (IL-6) promote insulin resistance in key metabolic tissues such as the liver, muscle, and adipose tissue (30, 31).

Conditions associated with elevated levels of insulin-antagonistic hormones—such as chronic hyperglycemia, pregnancy, polycystic ovary syndrome (PCOS), Cushing’s syndrome, acromegaly, and critical illness—can significantly aggravate IR. These physiological and pathological states are characterized by increased secretion of hormones such as glucagon, cortisol, growth hormone, catecholamines, and placental lactogen, which counteract the action of insulin. The resulting hormonal imbalance disrupts metabolic homeostasis by promoting inflammation, impairing insulin receptor signaling, and altering glucose and lipid metabolism, particularly in the liver, adipose tissue, and skeletal muscle. This ultimately reduces peripheral insulin sensitivity and increases the risk of developing metabolic disorders, such as type 2 diabetes mellitus (32–35).

A range of pharmacological agents has also been implicated in the development of insulin resistance. Glucocorticoids, statins, antipsychotics, immunosuppressants, and protease inhibitors impair insulin sensitivity by interfering with insulin signaling, generating oxidative stress, or modulating metabolic pathways (33).

Additionally, aging is associated with reduced insulin secretion, impaired glucose tolerance, and a progressive decline in insulin sensitivity. Age-related factors, including sarcopenia, central adiposity, mitochondrial dysfunction, and oxidative stress, contribute to these effects. Reduced mitochondrial β-oxidation and lower glycolytic enzyme levels in muscle and liver tissues further exacerbate IR in elderly individuals (36, 37).

Table 2 provides a concise overview of various health and lifestyle factors contributing to IR’s development. These include central obesity, the impact of diseases, medications, and aging on IR. It highlights the interplay between metabolic changes, such as chronic hyperglycemia, and the persistent use of specific drugs like glucocorticoids and statins. Additionally, it lists physiological changes associated with aging, including mitochondrial dysfunction and increased adiposity.

**Table 2:** The most common environmental factors and their associations with the development of insulin resistance.

Factor	Description	Impact on insulin resistance (IR)	Reference
Obesity	Central obesity-induced IR is characterized by impaired insulin function. It affects glucose uptake and hepatic glucose output. Overweight and morbid obesity impact insulin sensitivity.	Massive adipose tissue accumulation leads to systemic IR through endocrine dysregulation and inflammation. Elevated free fatty acids (FFA) and proinflammatory factors, such as TNF-α and IL-6, contribute to IR in the liver, muscle, and adipose tissue.	(30, 31)
Diseases as chronic hyperglycemia, pregnancy, polycystic ovary syndrome (PCOS), Cushing’s syndrome, acromegaly, and critical illness	Chronic hyperglycemia, high free fatty acidemia, pregnancy, and increased levels of insulin-antagonistic hormones contribute to insulin resistance.	These conditions promote IR via increased inflammation, hormonal imbalance, and altered glucose and lipid metabolism.	(32–35)
Drugs and Medications	Several medications, including glucocorticoids, statins, antipsychotics, immunosuppressants, and protease inhibitors, are linked to the development of insulin resistance.	These drugs interfere with insulin signaling pathways, induce oxidative stress, or modulate glucose and lipid metabolism, leading to impaired insulin sensitivity and glucose intolerance.	(33)
Aging	Aging leads to reduced insulin secretion, decreased glucose tolerance, and increased IR. Aging-associated changes, such as sarcopenia, excess adiposity, osteoporosis, central obesity, oxidative stress, mitochondrial dysfunction, and altered metabolism, occur in both muscle and liver tissues.	The prevalence of IR and T2DM increases with age, influenced by the accumulation of visceral fat, oxidative stress, mitochondrial dysfunction, and metabolic changes. Reduced mitochondrial β-oxidation and glycolytic protein levels in aged individuals contribute IR.	(36, 37)

### Cellular organelle interactions and vesicle-mediated communication in stress responses: implications for the development of insulin resistance

The complex interactions between cellular organelles are essential for maintaining metabolic homeostasis, and their dysregulation plays a crucial role in the development of IR. One of the key organelles involved is the endoplasmic reticulum (ER), which is responsible for protein folding and calcium (Ca^2+^) homeostasis. When overwhelmed by misfolded proteins or calcium imbalance, the ER initiates the unfolded protein response (UPR), a stress signaling cascade. Chronic ER stress activates inflammatory pathways, particularly the c-Jun N-terminal kinase (JNK) and inositol-requiring enzyme 1 (IRE1), which impair insulin receptor signaling and contribute to peripheral and hypothalamic insulin resistance (IR) (38, 39).

Mitochondria, as the primary site of oxidative phosphorylation and ATP synthesis, are crucial for energy-intensive processes such as insulin-stimulated glucose uptake. Impaired mitochondrial function leads to decreased ATP availability and reduced metabolic flexibility in insulin-responsive tissues, such as skeletal muscle and the liver, thereby promoting systemic IR (40). Furthermore, mitochondrial dysfunction is often accompanied by increased production of reactive oxygen species (ROS). When the antioxidant regulatory system, particularly involving PGC-1α, is overwhelmed, ROS accumulation interferes with insulin receptor substrate (IRS) phosphorylation and amplifies inflammatory signaling, thereby further exacerbating insulin resistance, especially in the contexts of aging and metabolic overload (40). A closely related structure, the mitochondria-associated membrane (MAM), forms contact sites between the ER and mitochondria, playing a critical role in lipid transfer, calcium exchange, and the coordination of stress signaling. Dysfunctional MAMs impair these processes, triggering ER stress and mitochondrial dysfunction, especially in the hypothalamus, where they have been implicated in the pathogenesis of central insulin resistance (38).

Additionally, vesicle trafficking within the ER-endomembrane system, which involves transport between the ER, Golgi apparatus, and plasma membrane, is crucial for the proper localization of insulin receptors and glucose transporter 4 (GLUT4). This process relies on coat protein complexes (COPI/COPII) and SNARE-mediated vesicle fusion. Disruptions in vesicular transport can hinder insulin receptor recycling and GLUT4 translocation, thereby contributing to peripheral insulin resistance (39).

Moreover, exosome-like vesicles (ELVs), which are secreted from various cells and contain biologically active cargo such as proteins, lipids, and RNAs, serve as mediators of intercellular communication. Pathological ELVs derived from inflamed or metabolically stressed cells can impair pancreatic β-cell function or induce IR in distant tissues by delivering inflammatory or inhibitory signals (41).

Finally, broad organelle crosstalk, including dynamic interactions among the ER, mitochondria, lysosomes, and peroxisomes, is essential for nutrient sensing, redox regulation, and metabolic adaptation. Disruption of this crosstalk impairs the cellular response to metabolic demands and stress, thereby fostering the progression of insulin resistance (39). These organelle-level disruptions represent converging mechanisms that underlie the complex pathophysiology of IR across different tissues and systems.

Table 3 summarizes the fundamental roles of subcellular organelle interactions in maintaining cellular homeostasis and details how imbalances in these interactions contribute to the development of insulin resistance.

**Table 3:** The subcellular organelles dysfunction and its associations with the development of insulin resistance.

Organelle interaction	Mechanism/process	Impact on insulin resistance (IR)	Reference
Endoplasmic reticulum (ER)	Protein folding, Ca^2+^ homeostasis, Unfolded Protein Response (UPR), ER stress	ER stress activates inflammatory signaling (e.g., JNK, IRE1), impairs insulin receptor signaling, and contributes to peripheral and hypothalamic insulin resistance.	(38, 39)
Mitochondria	β-oxidation, oxidative phosphorylation, ATP synthesis	Mitochondrial dysfunction decreases ATP production, impairs insulin-stimulated glucose uptake in muscle and liver, and contributes to systemic insulin resistance.	(40)
Mitochondria-associated membranes (MAMs)	Membrane contact sites (MCSs), lipid and Ca^2+^ exchange, UPR-mitochondrial crosstalk	MAM dysfunction disrupts Ca^2+^ signaling and mitochondrial energetics, induces ER stress, and has been implicated in central insulin resistance, such as in the hypothalamus.	(38)
Mitochondrial ROS production	Regulated by PGC-1α and antioxidant systems	Excess ROS impairs insulin receptor substrate (IRS) signaling and promotes inflammation, leading to reduced insulin sensitivity, particularly in the context of aging or metabolic overload.	(40)
Vesicle trafficking (ER-endomembrane system)	Involves the ER, Golgi, and plasma membrane via COPII/COPI vesicles and the Soluble N-ethylmaleimide-sensitive factor Attachment protein REceptor (SNARE) complexes	Disruption in vesicle transport affects insulin receptor trafficking and GLUT4 recycling, contributing to peripheral insulin resistance.	(39)
Exosome-like vesicles (ELVs)	Secreted from various cells, cargo includes proteins, lipids, RNAs	Alter insulin signaling via intercellular communication; pathogenic ELVs impair β-cell function or promote systemic insulin resistance.	(41)
Organelle crosstalk (general)	Includes ER, mitochondria, lysosomes, and peroxisomes	Disruption in dynamic organelle interaction impairs nutrient sensing, redox balance, and metabolic adaptation, fostering insulin resistance.	(39)

### The intracellular stressors that contribute to the development of insulin resistance

Insulin resistance arises from complex interactions among inflammatory, metabolic, and cellular stress pathways. Key contributors include the activation of inflammatory mediators such as NF-κB, JNK, and TLR4, which impair insulin receptor substrate (IRS) signaling (42, 43). Immune cells, such as M1 macrophages and CD8+ T cells, exacerbate inflammation, whereas Tregs and M2 macrophages can enhance insulin sensitivity (44, 45). Hypoxia in adipose tissue promotes IR via HIF-1α-induced inflammation and ceramide accumulation, although muscle hypoxia may enhance GLUT4 translocation (3, 46). Lipotoxicity, triggered by excessive free fatty acids, leads to ER stress, oxidative stress, and β-cell dysfunction (47, 48). High levels of ROS activate stress kinases that disrupt insulin signaling (49, 50). Genotoxic and mitochondrial stress amplify dysfunction (51), while impaired autophagy worsens ER stress and FGF21 resistance (52, 53).

Table 4 summarizes how factors such as inflammation, hypoxia, lipotoxicity, oxidative stress, genotoxic stress, and dysregulation of apoptosis and autophagy impact key metabolic target tissues, thereby impairing the normal metabolic functions of insulin. Table 4 briefly describes the mechanisms through which these influences employ their effects, including the roles of immune cells, inflammatory pathways, and the direct impact of lipid metabolites on insulin signaling pathways.

**Table 4:** The role of intracellular stress factor pathways in the progression of insulin resistance.

Factor	Description	Impact on insulin resistance (IR)	Key mediators	Molecular mechanism	Reference
Inflammatory pathway	Increased activity of IKKβ/NF-κB and JNK1 in adipose and liver; TLR4 promotes inflammatory mediator expression.	Activates inflammatory factors that inhibit IRS tyrosine phosphorylation, disrupt insulin signaling, and increase circulating FGF21.	NF-κB, JNK, TLR4, FGF21, RANKL	TLR4–NF-κB/JNK pathways activate cytokines → inhibit IRS-1/2 → disrupt PI3K-Akt signaling → IR. FGF21 dysregulation impacts systemic metabolism.	(42, 43)
Immunocytes	Roles of macrophages, neutrophils, mast cells, NK, CD4/CD8 T cells, Tregs, B cells in inflammation and IR.	M1 macrophages exacerbate IR through cytokines, whereas M2 macrophages and eosinophils improve insulin sensitivity. T/B cells influence glucose homeostasis.	IL-1β, TNF-α, IFN-γ, IL-10, CD11c, CD206, MCP-1	Inflammatory immune cell infiltration → cytokine release → JNK/NF-κB activation → IRS inhibition. Regulatory cells modulate the balance between inflammation and glucose metabolism.	(44, 45)
Hypoxia	Hypoxia in adipose tissue (obesity, HFD) and intermittent hypoxia (e.g., OSA).	Promotes IR via the production of inflammatory mediators and ceramides. Paradoxically, in muscle, hypoxia may stimulate the translocation of GLUT4.	HIF-1α, ceramide, TNF-α, IL-6	HIF-1α activation → transcription of inflammatory genes and ceramide synthesis → inhibits insulin signaling; AMPK activation in muscle → GLUT4 translocation (protective).	(3, 46)
Lipotoxicity	Lipid spillover into non-adipose tissues; FFAs disrupt β-cell function and induce endoplasmic reticulum (ER) stress.	Causes IR via oxidative stress, β-cell dysfunction, and impaired insulin biosynthesis.	FFAs, ceramide, TNF-α, ROS	Excess FFAs → ER stress + ROS → JNK activation + mitochondrial dysfunction → IRS impairment and reduced insulin secretion.	(15, 47, 48)
Oxidative stress	Biomolecular modifications via ROS (e.g., hydroperoxidation, sulfonation).	Low ROS promotes insulin signaling, while high H2O2 activates JNK and inhibits IRS, leading to insulin resistance (IR).	ROS, H2O2, protein hydroperoxides, PTP1B	ROS at high levels → oxidative stress → JNK activation → inhibits insulin receptor/IRS phosphorylation → IR; mild ROS inhibits PTP1B → enhances insulin signaling.	(49, 50)
Genotoxic stress	DNA damage resulting from oxidative and mitochondrial stress triggers ER stress and apoptosis.	Promotes IR via mitochondrial dysfunction and disrupted insulin signaling. DNA repair can mitigate this.	8-OHdG, ROS, mtDNA mutations	DNA/mtDNA damage → ROS/ER stress → JNK activation → inhibits IRS and Akt signaling; apoptosis contributes to metabolic dysfunction.	(51, 115)
Apoptosis	Induced by oxidative and ER stress; mediated via mitochondrial and lysosomal pathways.	Apoptosis in insulin-sensitive tissues impairs metabolic regulation and contributes to IR.	Fas ligand, caspases, cytochrome c	Stress signals (oxidative/ER) → Fas–FasL or mitochondrial cytochrome c release → caspase cascade → tissue dysfunction → IR.	(116)
Autophagy dysregulation	Autophagy is inhibited in obesity due to oxidative stress and impaired lysosomal function.	Impaired autophagy increases ER stress and disrupts insulin signaling, contributing to IR. Resistance to FGF21 is linked to autophagy dysregulation.	LC3-II, p62/SQSTM1, LAMP2A, FGF21, ATG proteins	Inhibition of autophagy → accumulation of damaged organelles and proteins → ER stress → impaired insulin signaling; reduced FGF21 receptor signaling decreases the adaptive response to metabolic stress.	(52, 53)

### Endoplasmic reticulum stress and insulin resistance

As mentioned above, the ER is a central organelle in cellular physiology, playing a pivotal role in protein synthesis, lipid metabolism, and calcium storage and signaling. It is crucial for maintaining cellular function and survival under both normal and stressful conditions (54). The ER extends throughout the cytoplasm, consisting of the rough ER (rER) and smooth ER (sER). The rER, studded with ribosomes, is where the synthesis of membrane-bound and secretory proteins takes place. Meanwhile, sER lacks ribosomes and is involved in lipid synthesis, metabolism, and detoxification processes (55). The ER is responsible for the proper folding and post-translational modifications of proteins, ensuring they achieve their correct three-dimensional structure and function properly. Additionally, the ER regulates intracellular calcium levels, which are critical for numerous cellular processes, including muscle contraction, neurotransmitter release, and cell death (56).

The ER is a dynamic cellular organelle whose complex activities are influenced by various internal and external factors, including low oxygen (hypoxia) or hypoglycemia levels, elevated temperature, acidosis, alterations in calcium concentration, the redox environment, and energy availability (57). These effectors can disrupt the ER’s normal functions, leading to ER stress and affecting the protein folding process within its lumen (58). Protein folding depends on the interactions among chaperone proteins, foldases, and glycosylating enzymes alongside suitable calcium levels and an oxidizing environment (59). When ER stress occurs, the protein folding process is disrupted, resulting in the accumulation of unfolded or misfolded proteins. This ER stress triggers the unfolded protein response (UPR), a cellular mechanism and hallmark of ER stress (60).

The UPR is a cellular adaptive mechanism triggered by the accumulation of misfolded or unfolded proteins within the ER. Under physiological conditions, the UPR restores ER homeostasis by attenuating protein synthesis, upregulating molecular chaperones, and enhancing the degradation of misfolded proteins (61, 62). Chronic activation of the UPR is a common pathology in obesity and metabolic stress (63). The chronic activation of the UPR can induce inflammation, oxidative stress, and IR (64). The UPR is mediated through three major ER stress sensors: IRE1 (inositol-requiring enzyme 1), PERK (protein kinase RNA-like ER kinase), and ATF6 (activating transcription factor 6). IRE1 can activate JNK, which phosphorylates insulin receptor substrates (IRS) on serine residues, impairing insulin signaling. PERK phosphorylates eIF2α, upregulating ATF4 and CHOP, which contribute to apoptosis and β-cell dysfunction. ATF6 enhances ER chaperone expression but may also promote inflammation under prolonged stress. UPR also interacts with mitochondrial dysfunction and inflammatory pathways, amplifying metabolic derangements. Thus, while initially protective, prolonged UPR activation contributes to the development of systemic insulin resistance (65, 66).

The accumulation of unfolded, improperly folded, insoluble, and damaged proteins leads to marked impairment of cellular functions alongside proteotoxic effects that threaten cell survival. Cells defend themselves through the UPR, which plays a fundamental role in preserving intact proteins that are correctly folded and processed, thereby alleviating this threat (67). When terminally misfolded and irreparable, proteins are eliminated from the cell through one of two processes. The first is ER-associated degradation (ERAD), which transports these damaged proteins into the cytoplasm for breakdown and elimination by the proteasome. The second mechanism, aggresomal formation, occurs. This mechanism includes accumulating the damaged proteins and cellular waste into juxtanuclear complexes for recycling via autophagy (68). Closely linking autophagy to ER stress is a process known as ER-phage (69, 70).

As previously noted, the ER collaborates with other subcellular organelles and plays a crucial role in protein and lipid metabolism, steroid biosynthesis, and calcium homeostasis, in addition to its primary metabolic function of protein folding. Impairment in these processes results in perturbed ER homeostasis, commonly referred to as ER stress (71). Insulin resistance can serve as both a cause and consequence of an unchecked ER stress response (72). Table 5 illustrates the various mechanisms through which insulin resistance and endoplasmic reticulum stress are interconnected.

**Table 5:** The interplay between ER stress and insulin resistance.

Endoplasmic reticulum stress-related disorder	Key mediators	Mechanism of ER stress and IR	Reference
Impaired ER-mitochondria transport	PDK4, ITPR1, HSPA9, VDAC1, MAMs	ER stress is linked to IR through the regulation of ER-mitochondria Ca^2+^ transport and homeostasis. Activation of the PDK4 complex in obese mice promotes the formation and stabilization of mitochondria-associated membranes (MAMs), leading to mitochondrial Ca^2+^ overload, ER stress, and IR.	(117)
Disordered signaling pathways	MAPK8 (JNK1), IRS1	IR-induced ER stress leads to MAPK8 hyperactivation, which phosphorylates and inhibits IRS-1, resulting in systemic insulin resistance. ER stress-alleviators reduce hyperglycemia and systemic IR in obese diabetic mice.	(118)
Disrupted macrophages and immune cells function and inflammatory response	Macrophages, NK cells, SPP1, DDIT3, ATF4, EIF2AK3, eIF2α	Activation of immune cells in adipose tissues and liver contributes to IR progression through ER stress induction. This is evidenced by activated macrophages and NK cells promoting the secretion of proinflammatory cytokines, thereby aggravating ER stress and insulin resistance. Elevated levels of Secreted Phosphoprotein 1/Osteopontin (SPP1) upregulate ER stress markers, triggering insulin resistance. SPP1 promotes macrophage infiltration and the M1 inflammatory phenotype, leading to elevated cytokine levels (e.g., TNF-α, IL-6), which interfere with insulin signaling.	(72) (119)
Adiponectin and ER stress	Adiponectin, CHOP, ATF3, DsbA-L, Adiponectin receptors	Obesity-related hypoxic environments in adipocytes lead to ER stress, which downregulates adiponectin, contributing to insulin resistance. ER stress also degrades adiponectin, affecting its multimerization and stability, and further impairs its anti-inflammatory and insulin-sensitizing functions.	(120) (121)
Leptin, ER stress, and leptin resistance	Leptin, CHOP, C/EBPα, Mitofusin 2	ER stress downregulates leptin expression and inhibits leptin receptor signaling, contributing to leptin resistance, increased appetite, and reduced energy expenditure. This resistance is associated with obesity-induced ER stress and the dysfunction of the mitochondria-ER interaction.	(122, 123)
Disordered expression of ER stress response genes	ob/ob mice, Xbp1, ORP150	In mouse models, ER stress is associated with obesity and insulin resistance, as observed in ob/ob mice and Xbp1 knockout (KO) mice. The increased expression of ER stress response genes and the molecular chaperone ORP150 in obese patients and models suggests a significant role of ER stress in these conditions.	(124)
Autophagy dysregulation	Autophagosomes, Atg proteins, ROS, PPAR-α, GSK3β, Atg7, FGF21	IR is associated with inhibited autophagy due to oxidative stress. Inhibited autophagy in adipose tissue contributes to insulin resistance and ER stress	(64)
Dysregulation of the antioxidant response	Nrf2, Keap1, ROS, p53, ERK, GSK3β, cortisol, 11βHSD1, FGF21	Nrf2 is a critical regulator of antioxidant responses, but its activity is suppressed in IR, leading to increased oxidative and ER stress.	(64)

### The protective role of LCHF-KD

The ketogenic diet (KD) was introduced in the 1920s as a treatment for epilepsy. The KD is characterized by its low carbohydrate content, high fat content, and moderate protein content, aligning with the principles of fasting. Over the last decades, the application has expanded to include managing obesity and achieving quick weight loss. It has been investigated for potential other health issues such as type 2 diabetes, non-alcoholic fatty liver disease, various cancers, Alzheimer’s disease, cardiovascular diseases, chronic kidney diseases, and during pregnancy (73).

This diet regimen aims to achieve physiological ketosis, utilizing ketones as an alternative energy source to glucose. There are multiple variations of the KD, all sharing the common goal of markedly limiting carbohydrate intake. A standard ketogenic diet typically involves consuming under 50 g of carbohydrates daily, approximately 75% dietary fat, and protein intake between 1 and 1.4 g per kilogram of body weight (74). Therefore, it is referred to as a low-carbohydrate, high-fat ketogenic diet (LCHF-KD).

Restricting carbohydrate intake, which is normally the primary energy source for body tissues, leads to reduced insulin production, decreased fat storage, and increased glucose production through the process of gluconeogenesis. However, this glucose production is insufficient for the body’s needs, causing a shift in the primary energy source to fat breakdown and the production of ketone bodies (KBs) in the liver, which is the main target of the LCHF-KD. These ketone bodies can also cross the blood–brain barrier to fuel the central nervous system (21, 73, 75).

LCHF-KD also enhances fat metabolism in muscles, even in endurance athletes. Over the past four decades, various adaptations and strategies involving LCHF have been studied, including both ketogenic and non-ketogenic forms. Since 2012, research has increasingly examined the potential benefits of ketogenic LCHF-KD on endurance performance, noting significant metabolic changes and suggesting that these diets can maximize fat oxidation rates and increase ketone production, thereby providing additional energy sources for muscles and the central nervous system (74, 76).

We discussed the physiological effects of LCHF-KD in detail and published the findings in 2022 (73). The LCHF-KD exerts multiple beneficial effects across metabolic, inflammatory, mitochondrial, and epigenetic pathways. One of its primary effects is the improvement of insulin sensitivity, achieved through carbohydrate restriction, which lowers insulin secretion and mitigates hyperinsulinemia, a key driver of insulin resistance (73, 77). By stabilizing blood glucose levels, LCHF-KD also contributes to the inhibition of protein glycation. This effect reduces the formation of advanced glycation end products (AGEs), harmful compounds implicated in diabetes-related vascular complications and atherosclerosis (73, 78).

At the mitochondrial level, LCHF-KD ameliorates dysfunction and oxidative stress by maintaining mitochondrial membrane potential and optimizing the efficiency of the electron transport chain (ETC). It also decreases the generation of reactive oxygen species (ROS) and upregulates the Nrf2 antioxidant pathway, leading to enhanced expression of detoxifying enzymes (73, 79).

A major contributor to the anti-inflammatory effect of the ketogenic diet is β-hydroxybutyrate (ßOHB), a ketone body that inhibits inflammasome activation and crosses the blood–brain barrier to activate the HCA2 receptor, thereby reducing neuroinflammation and providing neuroprotection (73, 80). Emerging evidence also supports its role in fighting malignancy-associated features. By restricting glucose availability, LCHF-KD disrupts the Warburg effect, thereby inhibiting tumor growth. Furthermore, it enhances the efficacy of PI3K inhibitors by lowering insulin levels and preventing the reactivation of oncogenic pathways (73, 81).

In lipid metabolism, the LCHF-KD shows promise in preventing dyslipidemia by lowering triglyceride levels and shifting LDL-C particles toward larger, less atherogenic forms while maintaining or increasing HDL-C levels (73, 82).

On a molecular level, LCHF-KD impacts the epigenome by elevating intracellular adenosine levels, which inhibit DNA methylation and histone deacetylases (HDACs). This results in increased histone acetylation and modulation of gene expression, contributing to long-term metabolic and protective effects (73, 83). Moreover, the LCHF-KD diet has a positive impact on the gut microbiota, promoting beneficial species such as Akkermansia and reducing pro-inflammatory microbes. These changes reinforce the intestinal barrier, lower systemic inflammation, and improve metabolic outcomes such as insulin sensitivity (73, 84, 85).

Overall, LCHF-KD emerges as a multifaceted intervention with systemic effects that extend beyond weight loss, encompassing metabolic regulation, cellular protection, and epigenetic modulation, as summarized in Table 6. This table presents the metabolic impact of LCHF-KD and its mechanisms.

**Table 6:** The physiological and metabolic effects of the LCHF-KD.

Effects	Key mediators	Mechanisms	Reference
Improve insulin sensitivity	Carbohydrate restriction, Insulin	LCHF-KD limits carbohydrate intake, reducing insulin production and improving insulin sensitivity.	(73, 77)
Inhibition of protein glycation	Blood glucose stabilization, AGEs	By stabilizing blood glucose levels and reducing spikes, LCHF-KD lowers the formation of advanced glycation end products (AGEs) in proteins, which can otherwise lead to complications such as atherosclerosis and diabetes.	(73, 78)
Inhibiting mitochondrial dysfunction & oxidative stress	Mitochondria, ROS, Nrf2 pathway	LCHF-KD enhances mitochondrial functions by maintaining electrochemical transmembrane potential and optimizing the efficiency of the electron transport chain (ETC). LCHF-KD reduces ROS production and increases the activation of the Nrf2 antioxidant pathway, enhancing the body’s detoxification genes.	(73, 79)
Anti-inflammatory effects	β-hydroxybutyrate (ßOHB), HCA2 receptor, inflammasomes	LCHF-KD generates ßOHB, which suppresses the activation of stress-related inflammasomes. ßOHB also facilitates crossing the blood–brain barrier to reduce neuroinflammation by activating the HCA2 receptor, which has neuroprotective and anti-inflammatory effects.	(73, 80)
Fighting malignancy-associated features	Glucose restriction, Warburg effect, PI3K inhibitors	LCHF-KD restricts glucose, impacting cancer cell metabolism (the Warburg effect) and inhibiting tumor growth. It enhances the efficacy of PI3K inhibitors by reducing hyperinsulinemia, which can reactivate cancer pathways.	(73, 81)
Prevention of dyslipidemia	Lipid profiles, triglycerides, LDL, HDL	LCHF-KD reduces triglyceride levels and may affect LDL particle size, promoting less atherogenic forms.	(73, 82)
Effects on the epigenome	DNA methylation, histone acetylation	LCHF-KD influences epigenetic changes through increased adenosine levels, which block DNA methylation and inhibit histone deacetylases (HDACs), leading to increased histone acetylation and affecting gene expression linked to metabolism and disease prevention.	(73, 83)
Gut microbiota	Gut microbiome composition, intestinal barrier	LCHF-KD alters the composition of the gut microbiota, increasing beneficial bacteria, such as Akkermansia, and decreasing inflammatory bacteria. These changes can protect the intestinal barrier, reduce inflammation, and enhance insulin sensitivity.	(73, 84, 85)

### Experimental evidence on the impact of LCHF-KD on insulin resistance-related ER stress

The KD, characterized by its LCHF composition, has been extensively studied across various health contexts, including its impact on metabolic diseases, neuroprotection, and ER stress (86).

Lei et al. (87) have provided insights into how nutritional ketosis influences ER stress and its effects on insulin signaling in the liver. During early lactation, a period of negative energy balance, dairy cows often exhibit elevated levels of β-hydroxybutyrate (β-OHB) and fatty acids, indicative of ketosis. Their study highlights that many markers exhibit a link between insulin resistance and increased activation of ER stress pathways, such as IRE1α, PERK, and ATF6. These findings are significant as they suggest a link between exacerbated ER stress and impaired liver function. Notably, the study demonstrates that inhibiting ER stress reverses these adverse effects, suggesting that targeting ER stress may mitigate hepatic insulin resistance (87) through nutritional ketosis.

In another study focusing on skeletal muscle, the effects of a KD on induced insulin resistance were analyzed, showing potential benefits in alleviating insulin resistance through ER stress modulation. In mouse muscle cells, KD reversed the activation of key ER stress markers (IRE1 and BIP) and enhanced cellular glucose uptake by improving insulin signaling. This mitigating effect was particularly evident in the restoration of the AKT/GSK3β pathway and the increase in Glut4 protein translocation to the cell membrane. These results suggest that the ketogenic diet affects liver health and skeletal muscle insulin sensitivity, at least in part, by modulating ER stress (88).

Furthermore, the KD’s role in neuroprotection, particularly concerning hypoglycemia-induced brain injury and stroke, has been demonstrated. In mice subjected to hypoglycemic conditions or stroke models, KD has been shown to exert protective effects by modifying gut microbiota, enhancing dendritic spine morphology, and reducing neuronal apoptosis. These neuroprotective effects were linked to the suppression of ER stress pathways like IRE1-XBP1 and ATF6. Additionally, in stroke models, KD inhibited the TXNIP/NLRP3 inflammasome pathway, a part of the innate immune response linked to inflammation and ER stress, further underscoring the diet’s potential to mitigate ER stress and protect against neurological damage (87).

To further elucidate the role of the KD in modulating ER stress, focused on its neuroprotective effects in stroke models, a particular study investigated the influence of KD on the nucleotide-binding domain (NOD)-like receptor protein 3 (NLRP3) inflammasome, which is a critical component in the inflammatory response and has been implicated in the pathogenesis of stroke (89). Their findings demonstrated that mice on KD exhibited increased resilience against the effects of stroke. Mechanistically, KD appeared to reduce the activation of the NLRP3 inflammasome in the brain. This reduction in NLRP3 inflammasome activity was associated with a decrease in ER stress induction. Notably, the study also highlighted the role of βOHB, a ketone body produced during ketosis, which inhibits mitochondrial dysfunction by preventing the mitochondrial translocation of dynamin-related protein 1 (Drp1)—a process involved in mitochondrial fission and a contributor to cellular stress. Additionally, βOHB’s role in suppressing ER stress-induced NLRP3 inflammasome activation emphasizes its potential as a therapeutic agent (89).

At the level of insulin resistance-related vascular pathology and endothelial dysfunction, the experimental study by Eldakhakhny et al. provides compelling evidence that a LCHF-KD diet can effectively ameliorate ERstress in the context of insulin resistance and endothelial dysfunction (86). Using a dexamethasone (DEX)-induced metabolic syndrome rat model, the study divided 40 male Sprague–Dawley rats into four groups: a control group, a DEX-treated group receiving a standard diet, a DEX-treated group receiving an LCHF-KD diet, and a DEX-treated group receiving a HCLF-KD diet. The DEX-treated animals exhibited clear signs of metabolic syndrome, including increased body mass index, insulin resistance (as evidenced by elevated HOMA-IR), and histological signs of aortic endothelial dysfunction, oxidative stress, and enhanced expression of ER stress markers, such as CHOP, PINK1, and BNIP3. Remarkably, animals in the DEX + LCHF-KD group showed significant improvements across all measured parameters. ER stress markers were downregulated to near-control levels, and ultrastructural analysis revealed the restoration of normal ER morphology, a reduction in autophagosomes, and preserved mitochondrial integrity (86). The study also demonstrated that the LCHF-KD diet decreased oxidative stress markers and normalized autophagy-related gene expression (p62, LC3, BECN-1), suggesting improved cellular homeostasis. These findings confirm that the LCHF-KD diet exerts a protective effect against insulin resistance-induced ER stress and endothelial dysfunction, underscoring its therapeutic potential as a non-pharmacological dietary strategy for managing metabolic syndrome and related vascular complications (86).

These recent studies strongly support the idea that the LCHF-KD has a beneficial role in managing ER stress across different biological systems. Whether by improving hepatic insulin sensitivity, enhancing muscle glucose uptake in insulin-resistant mice, protecting neural tissue from hypoglycemic damage and stroke, and mitigating vascular endothelial dysfunctions, LCHF-KD appears to modulate endoplasmic reticulum stress pathways effectively. These findings enhance our understanding of the ketogenic diet’s multifaceted roles in health and disease and highlight potential therapeutic targets for conditions associated with elevated endoplasmic reticulum stress. Thus, LCHF-KD offers a promising avenue for further research and therapeutic exploration.

### Understanding the role of the LCHF in mitigating stress-related insulin resistance

The LCHF-KD diet has demonstrated multiple protective roles in mitigating ER stress and improving insulin sensitivity through diverse molecular mechanisms. Firstly, it reduces lipotoxicity by enhancing lipid metabolism and decreasing glucotoxicity, which is achieved through the downregulation of SREBP-1c and upregulation of PPAR-α and CPT1, promoting β-oxidation and reducing ceramide synthesis and intracellular lipid accumulation, ultimately decreasing ER burden (90–92). The LCHF-KD diet also enhances insulin sensitivity through the protective effects of βOHB, which inhibits the NLRP3 inflammasome and JNK pathway and activates AMPK, thereby improving downstream insulin signaling (93). Its anti-inflammatory effects are mediated through the suppression of NF-κB and JNK pathways, reduction of TNF-α and IL-6, and inhibition of TLR4-mediated inflammatory responses, all of which contribute to improved IRS-1/Akt signaling (73, 80, 94). In terms of its antioxidant effects, the LCHF diet activates the Nrf2 pathway. It enhances the expression of antioxidant enzymes, such as superoxide dismutase (SOD) and catalase, thereby reducing mitochondrial ROS and lipid peroxidation and protecting the ERfrom oxidative damage (73, 95, 96). It also improves hormonal regulation, as lower insulin levels resulting from LCHF-KD intake diminish IRE1 and PERK activation, thereby maintaining ER homeostasis and reducing the accumulation of misfolded proteins (66). Additionally, the LCHF-KD diet enhances autophagy, which facilitates the clearance of misfolded proteins and damaged organelles from the ER. This effect is driven by AMPK activation and mTOR inhibition, along with the upregulation of autophagy-related proteins such as Atg5/7 and LC3-II (86). Moreover, the LCHF-KD modulates the gut microbiota by reducing the population of LPS-producing bacteria and increasing the population of microbes that produce short-chain fatty acids (SCFAs). Short-chain fatty acids (SCFAs) suppress histone deacetylases (HDACs) and inflammatory gene expression while reducing intestinal permeability, thereby limiting systemic inflammation and ER stress (97). Together, these interconnected mechanisms highlight the therapeutic potential of the LCHF-KD diet in addressing insulin resistance and ER stress-associated metabolic dysfunction.

The potential roles and the mechanisms by which a LCHF mitigates ER stress-related insulin resistance are presented in Table 7. Table 7 illustrates the diet’s impact on metabolic stability, inflammatory response, antioxidant capacity, and other factors, highlighting how these elements contribute to enhanced insulin sensitivity and cellular health.

**Table 7:** Mechanisms of the LCHF in alleviating ER stress-related insulin resistance.

The potential roles of the LCHF diet	Effect	Molecular mechanism	References
Reduction in lipotoxicity	Reduces ER stress by enhancing lipid metabolism and decreasing glucotoxicity, promoting metabolic stability	Downregulation of SREBP-1c and upregulation of PPAR-α and CPT1 enhance β-oxidation; reduction in ceramide synthesis and intracellular lipid accumulation reduces ER burden.	(90–92)
Enhancing insulin sensitivity	Reduces ER stress in peripheral tissues by leveraging the protective effects of ketone bodies, similar to those observed in neuronal cells	Ketone bodies (e.g., β-hydroxybutyrate) reduce oxidative and ER stress by inhibiting NLRP3 inflammasome and JNK pathway; activate AMPK → enhances insulin signaling.	(125)
Anti-inflammatory effects	Reduces cytokine-induced ER stress and improves insulin signaling by modulating inflammatory responses	Suppresses NF-κB and JNK signaling, reduces TNF-α, IL-6 expression; inhibits TLR4-mediated inflammatory response; improves IRS-1/Akt signaling.	(73, 80, 94)
Antioxidant effect	Decreases ER stress by reducing oxidative stress through enhanced mitochondrial efficiency and antioxidant enzyme activity	Enhances Nrf2 activation and antioxidant enzymes (SOD, catalase); reduces mitochondrial ROS and lipid peroxidation; protects against ER oxidation.	(73, 95, 96)
Improved hormonal regulation	Balances hormones like insulin, which can mitigate ER stress by improving glucose and lipid homeostasis	Lower insulin levels reduce IRE1 and PERK activation; improved insulin/IGF-1 signaling stabilizes ER homeostasis and prevents misfolded protein accumulation.	(98)
Enhanced autophagy	Facilitates the removal of misfolded proteins from the ER, thereby reducing ER load and stress	Activates autophagy via AMPK and inhibition of mTOR; increases Atg5/7 and LC3-II expression; promotes ER-to-lysosome protein degradation.	(86)
Modulation of gut microbiota	Affects gut-derived signals that influence systemic inflammation and ER stress, thereby impacting insulin resistance	LCHF reduces LPS-producing bacteria; increases SCFA-producing microbes; SCFAs inhibit histone deacetylases (HDACs) and inflammatory gene expression; lowers gut permeability → less systemic ER stress.	(97)

## Discussion

The increasing prevalence of metabolic diseases has encouraged the evaluation of dietary interventions as potential therapeutic strategies. Among these, the LCHF-KD has garnered attention for its role in modulating ER stress and mitigating insulin resistance, suggesting its potential applicability in personalized medicine.

According to the mechanisms mentioned above, the LCHF-KD could mitigate ER stress-related insulin resistance by inhibiting lipotoxicity (92), increasing insulin sensitivity with concordant prevention of insulin resistance (21), having anti-inflammatory Effects (94), the antioxidant effect (96), improving hormonal balance (98), augmenting autophagy (86), and modulating the gut microbiota population (97).

The potential of the LCHF-KD in personalized medicine has gained attention (99). The intervention of the LCHF-KD in personalized medicine depends on its compliance with the metabolic states and genetic backgrounds of each individual (100). Accordingly, healthcare providers can adapt the LCHF-KD to optimize its impact on ER stress and insulin sensitivity by evaluating a subject’s genetic predisposition to insulin resistance and metabolic profile. The role of the LCHF-KD within this framework is particularly convincing due to its direct impact on metabolic processes related to endoplasmic reticulum stress and insulin resistance.

Personalized medicine studies the genetic variations influencing an individual’s response to the LCHF-KD (101). Genes involved in various metabolic and inflammatory pathways, such as fatty acid metabolism (FTO, APOE), insulin secretion (TCF7L2, IRS1), and inflammation (TNFα, IL6), play significant roles in determining dietary efficacy and individual adaptations. Consequently, genetic variations affecting fat metabolism require modifications in dietary fat types or amounts to optimize health outcomes and prevent undesirable LCHF-KD-related effects (101, 102).

To maximize the benefits of LCHF-KD interventions, healthcare providers must utilize comprehensive metabolic profiling, including assessments of glucose metabolism, lipid levels, and inflammatory markers, both in the short term (103) and long term (104). This understanding enables the personalization of nutritional recommendations based on genetic predispositions and metabolic states. For example, introducing omega-3 supplements with antioxidant effects can be specifically recommended to those who have observed increases in oxidative stress or inflammation, ensuring the diet’s effectiveness and safety (93). Furthermore, gut microbiome composition is a crucial consideration in contemporary dietary planning. The microbiota’s composition, population, and roles in metabolizing fats and carbohydrates significantly determine the outcomes of an LCHF-KD (105). Therefore, personalizing diet plans based on microbiome analysis could enhance insulin sensitivity and reduce ERstress, providing a targeted approach to managing metabolic diseases.

To further illustrate the cumulative findings across experimental and clinical studies, Table 8 summarizes the available evidence demonstrating the modulatory effects of the LCHF-KD on ER stress and insulin resistance. The table integrates data from *in vivo* experimental animal and human clinical studies, highlighting how dietary-induced ketosis influences ER-stress signaling pathways and insulin sensitivity through molecular, metabolic, and anti-inflammatory mechanisms.

**Table 8:** Summary of experimental and clinical evidence on the effect of low-carbohydrate, high-fat ketogenic diet (LCHF-KD) on endoplasmic reticulum (ER) stress and insulin resistance.

Study	Year	Model or population	Diet composition (approx.)	ER-stress biomarkers	Insulin-resistance outcomes	Principal mechanism(s) implicated	Study type
Paoli et al. (20)	2023	Adults with overweight/T2DM (review of trials)	<10% carbohydrates, 70–75% fat, 15–20% protein	ER-stress biomarkers are not directly measured	↓ HOMA-IR, improved glycemic control	↓ Hyperinsulinemia, ↓ glucotoxicity; indirect ER-stress relief	Clinical [Human review (systematic review of human studies)]
Jeziorek et al. (17)	2022	Lipedema patients [Women (*n* = 91) with Lipedema (bilateral sub-cutaneous fat accumulation)]	LCHF vs. moderate-CHO/fat; LCHF: <10% CHO, 70–75% fat, 15–20% protein for 16 weeks	Not reported	Improved body composition and metabolic parameters	↓ Insulin secretion and load; inferred ↓ ER burden	Clinical Human intervention
Eldakhakhny et al. (86)	2024	Dexamethasone-induced metabolic syndrome rats	Low-carb, high-fat vs. high-carb, low-fat	↓ CHOP, ↓ PINK1, ↓ BNIP3, normalized ER ultrastructure	↓ HOMA-IR, restored endothelial function	↓ ER stress, ↑ autophagy, ↓ oxidative stress	In vivo/Exprimental animal study
Bima et al. (21)	2023	Brain insulin resistance in Dexamethasone-induced metabolic syndrome rats	LCHF-KD	↓ ER-stress signaling markers; improved neuronal insulin signaling	Improved brain insulin sensitivity	Anti-inflammatory and antioxidant effects of ketone bodies	*In vivo*/Exprimental animal study
Li et al. (87)	2021	One-month-old male mice (hypoglycemia model induced by insulin)	Ketogenic diet: high-fat (≈90%), very low carbohydrate (≈1–2%), moderate protein (≈8%)	↓ IRE1–XBP1 and ATF6 pathways; ↓ GRP78, CHOP expression; ↓ neuronal apoptosis	Improved hepatic insulin signaling Improved hypoglycemia-induced neuroinjury	KD mitigated ER-stress-mediated apoptosis, improved hippocampal neurogenesis, restored synaptic plasticity, and modulated gut microbiota (Dorea ↑/Rikenella ↓)	*In vivo*/Exprimental animal study
Ma et al. (88)	2024	Mouse skeletal muscle cells (Mice fed high-fat diet → switched to ketogenic diet)	HFD to KD for 2 weeks (KD - high fat, very low carbohydrate)	↓ IRE1, ↓ BiP, restored AKT/GSK3β and GLUT4	Improved GTT, ITT, HOMA-IR; increased GLUT4 membrane translocation; restored Akt/GSK3β signaling	KD alleviated ER stress → enhanced insulin signaling via Akt/GSK3β pathway; increased GLUT4 translocation	*In vivo*/Exprimental animal study
Guo et al. (89)	2018	Mouse hypoglycemia and stroke models [Male C57BL/6 mice (4-week-old) + *in vitro* SH-SY5Y cells]	KD: high-fat, low-carbohydrate diet for 3 weeks (vs standard chow & high-carb diet groups)	↓ p-PERK-p-eIF2α-ATF4 pathway, ↓ CHOP expression in brain of MCAO mice (indicating reduced ER stress)	Reduced neuronal apoptosis and inflammation	KD suppressed ER stress and mitochondrial fission via ↓ Drp1 translocation → ↓ ROS → ↓ TXNIP/NLRP3 inflammasome activation → improved ischemic tolerance	*In vivo*/Exprimental animal study

Despite the potential for personalized LCHF-KD interventions in managing ER stress-related insulin resistance, it is of great significance. Still, several challenges arise when implementing LCHF-KD in the prevention and treatment of ER stress-related insulin resistance. These limitations and challenges include ensuring patient compliance, understanding long-term impacts, and integrating this approach within broader medical guidelines.

So, the current study recommends that to augment the therapeutic use of the LCHF-KD in personalized medicine for managing ER stress-related insulin resistance, Physicians and healthcare providers should receive dedicated training to customize the diet based on individual genetic and metabolic profiles. Collaborative care teams, including dieticians and physicians, should closely monitor and adjust patient diets and improve compliance. Education and awareness programs for the patient’s needs can enhance motivation and compliance. Furthermore, research should focus on identifying genetic markers and metabolic profiles that predict diet responsiveness, assessing long-term health impacts across diverse populations, and exploring the role of the gut microbiome.

## Conclusion

The relationship between ER stress and metabolic disease, particularly insulin resistance, is gaining increasing attention in the scientific community. Emerging research highlights the promising potential of targeting ER stress as a novel and effective strategy for preventing and managing insulin resistance and associated metabolic disorders. ER stress has been implicated in the development of insulin resistance, a pivotal element in numerous metabolic diseases, including T2DM, obesity, and cardiovascular diseases.

The potential of therapeutic agents that alleviate ER stress holds substantial promise. Targeting ER stress represents a cornerstone in the prevention and treatment of metabolic diseases. By integrating advanced therapeutic agents, strategic dietary interventions, and conventional therapies, we can enhance our approach to these health challenges, offering hope for better management and, ultimately, prevention of these conditions.

Dietary interventions also play a critical role in managing ER stress. LCHF-KD has been demonstrated to reduce ER stress markers in experimental models. These dietary components may exert their beneficial effects by modulating inflammation and oxidative stress, which are closely intertwined with ER stress. The LCHF-KD can diminish the ER’s overall burden, thereby improving insulin sensitivity and overall metabolic health.

Combining these novel therapeutic agents and dietary strategies with conventional therapies could revolutionize the management of metabolic diseases. By incorporating strategies aimed at reducing ER stress, it is possible to address one of the root causes of metabolic disorders, potentially improving therapeutic outcomes and reducing the incidence of disease progression.

Understanding the precise dietary factors that affect ER stress and insulin resistance, the mechanisms through which they exert their effects, and how these interact with individual genetic and lifestyle factors are critical areas for the personalized medicine approach.
